# Secondary syphilis presenting with alopecia and leukoderma in a stable HIV-positive patient in a resource-limited setting: a case report

**DOI:** 10.1186/s12981-024-00603-w

**Published:** 2024-04-01

**Authors:** Sukoluhle Khumalo, Yves Mafulu, Victor Williams, Normusa Musarapasi, Samson Haumba, Nkululeko Dube

**Affiliations:** 1 AIDS Healthcare Foundation, Manzini, Eswatini; 2 Centre for Global Health Practice and Impact, Georgetown University, Mbabane, Eswatini

**Keywords:** Secondary syphilis, Alopecia, Leukoderma syphilliticum, Human immuno-deficiency virus.

## Abstract

**Background:**

Syphilis is an infection caused by the bacteria T*reponema pallidum*. It is mainly transmitted through oral, vaginal and anal sex, in pregnancy and through blood transfusion. Syphilis develops in primary, secondary, latent and tertiary stages and presents with different clinical features at each stage. Infected patients can remain asymptomatic for several years and, without treatment, can, in extreme cases, manifest as damage in several organs and tissues, including the brain, nervous tissue, eyes, ear and soft tissues. In countries with a high human immunodeficiency virus (HIV) burden, syphilis increases the risk of HIV infections. We report the case of a young HIV-positive black woman who presented with alopecia and hypopigmentation as features of secondary syphilis.

**Case presentation:**

A virologically suppressed 29-year-old woman on Anti-retroviral Therapy (ART) presented with a short history of generalized hair loss associated with a non-itchy maculopapular rash and skin depigmentation on the feet. Limited laboratory testing confirmed a diagnosis of secondary syphilis. She was treated with Benzathine Penicillin 2.4MU. After receiving three doses of the recommended treatment, the presenting features cleared, and the patient recovered fully.

**Conclusion:**

This case demonstrates the importance of a high index of clinical suspicion and testing for syphilis in patients presenting with atypical clinical features of secondary syphilis, such as hair loss and hypopigmentation. It also highlights the challenges in diagnosing and clinically managing syphilis in a resource-limited setting.

## Background

Syphilis remains a public health threat, with an estimated seven million new cases in 2020 [1]. The disease burden is heavy in low- and middle-income countries, with a review of sexually transmitted infections (STIs) among pregnant women reporting a syphilis prevalence of 6.5% in Southern Africa [2]. A study conducted in Swaziland among 655 women aged 15 to 49 years reported the prevalence of syphilis to be 2.0% [3]. Morbidity from syphilis is related not only to the impact of the disease on individuals but also its role as a contributor to HIV acquisition, together with the morbidity and mortality associated with neonatal infections [4]. HIV – syphilis coinfection is considered a dangerous combination since HIV makes the failure of syphilis treatment more likely, and coinfection leads to more profound neurocognitive impairment [5, 6]. Conversely, syphilis increases susceptibility to HIV infection due to its negative impact on the immune system [4].

Syphilis’s natural history evolves with alternating episodes of clinical and immunologic stages [7]. It progresses in three classical stages – primary, secondary and latent [5, 8]. However, many presentations vary and do not follow the classical stages. A chancre characterises primary syphilis, extensive mucocutaneous lesions occur in secondary syphilis and latent syphilis is diagnosed on serologic testing with no symptoms [8, 9]. Secondary syphilis is characterised by generalised mucocutaneous lesions affecting the skin, mucous membranes, and, characteristically, the palms and soles [4]. The symptoms and signs of secondary syphilis spontaneously resolve within 3 to 12 weeks, even without treatment, and if left untreated, the patient enters the latent stage. An estimated 25% of untreated secondary syphilis cases symptomatically recur, mostly within 12 months [5, 10]. In this article, we report the case of a 29-year-old HIV-positive patient who presented with two distinctive features of secondary syphilis and how the condition was managed.

## Case presentation

A case report was compiled using information retrospectively collected from the patient’s electronic and physical medical records. Pictures were taken at different stages of the illness, with the patient’s verbal and written consent.

The patient is a 29-year-old HIV-positive female on a first-line dolutegravir (DTG)-based ART regimen. She is single and works in a clothing factory. She presented on March 23 2022, with a 2-week history of hair loss. She initially noted her braids falling off and then progressive hair loss on her scalp, eyebrows, eyelashes, axilla, and groin. There was mild associated itching with no scaling on the head. The patient was not on any new medications. She also complained of a non-itchy rash on the limbs, mainly on the hands, rough looking and brownish. She reported no contact with anyone with similar symptoms and has not been pregnant before.

On review of systems, she reported a painless pustule on the groin, which has been there for about a year, not growing, with no associated discomfort. She had not reported it before, as it was not causing any issues. She reported no other genitourinary symptoms.

The patient was diagnosed with HIV and commenced ART in 2014. She is still on first-line ART, initially initiated on TDF/3TC/EFV, and later transitioned to TDF/3TC/DTG on 30/12/2021 per HIV program recommendations, three months before presentation. Her latest laboratory results at presentation were a lower-than-detectable viral load (4/8/2021) and serum creatinine of 50 μm/L (4/8/2021).

### Examination

On physical examination, the patient looked well and stable; her temperature was 36.5^0^C, her blood pressure was 120/50mmHg, her heart rate was 80b/min, and her respiratory rate was 18c/min. She was pink, well hydrated, and had no jaundice or oedema. Her oral cavity was clear.

**Fig. 1:**
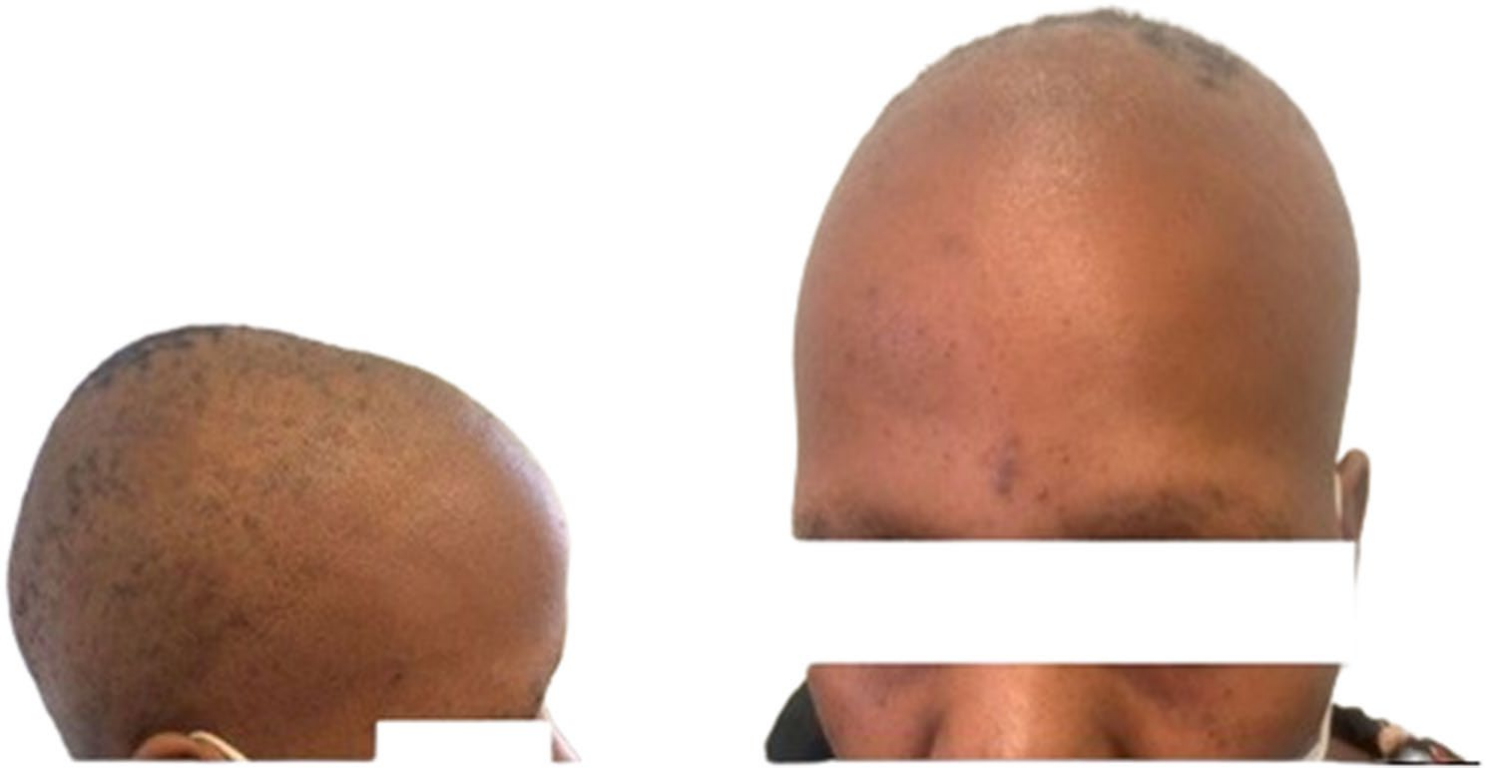
Extensive alopecia, with “moth-eaten appearance”, noted on the scalp, with patchy areas with hair. Hair loss is noted on the eyebrows.

**Fig. 2:**
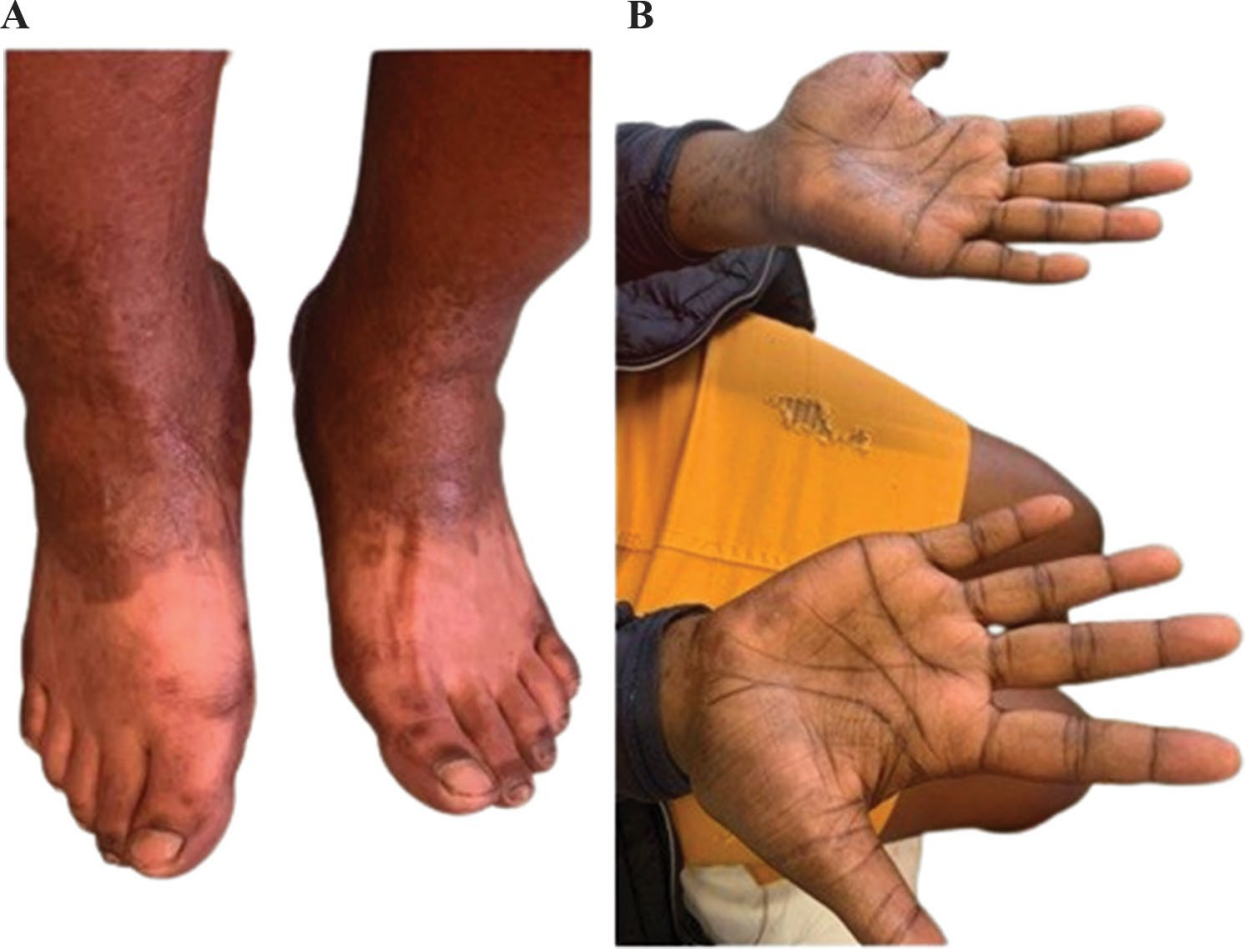
**A** Hypopigmentation of the anterior half of both feet, with a mottled appearance, consistent with *Leukoderma syphilliticum*, also called syphilis vitiligo. **B** Hyperpigmented maculopapular lesions in the palms of both hands also known as Buschke Ollendorf sign.

She had extensive scalp alopecia, with patchy areas with hair, but no scaling was noted. There was also hair loss on the eyebrows, eyelashes, axilla and groin regions (Fig. 1). She also had hyperpigmented papules on the palmar aspect of both hands extending to the wrist, also called Buschke Ollendorf sign when there is tenderness on the application of blunt pressure on the papules [11] (Fig. 2A) and asymmetrical, homogenous, clearly demarcated hypopigmentation on the anterior of both feet (Fig. 2B). On examination of her genitourinary system, she had a painless lump measuring about 0.5 cm x 0.5 cm on the left peri vulval region. There were no palpable lymph nodes in the groin on either side. Examination of her chest, cardiovascular system and abdomen were normal.

### Laboratory investigation and management

The investigations done for the patient at presentation included a full blood count (FBC) and a syphilis determine rapid tests. The full blood count showed a haemoglobin of 10.7 g/dl, mean corpuscular volume (MCV) of 90.3 fl., a white cell count of 4.1 × 10 ^9^cells/litre and a platelet count of 309 × 10^9^/litre. The syphilis determine rapid test (for antibodies) was positive. The titre was not estimated due to the limited laboratory capacity.

The patient was treated with benzathine penicillin 2.4MU stat per World Health Organization (WHO) guidelines for secondary syphilis [12]. At review after two weeks, some improvement was observed in the maculopapular rash on the palms, and the genital lump had disappeared completely. However, there was no change in hair growth and skin depigmentation. The patient missed her review two weeks later and only returned after three months for her ART refill. At this visit, she was attended to by another clinician and given another three-month supply of ART medication. Since her contacts were not updated in the database, she could not be reached for further review and evaluation until September 21 2022. The patient was given three doses of benzathine penicillin at this time, and a rapid plasma reagin (RPR) titre done in October 2022 was less than 1:8.

**Fig. 3:**
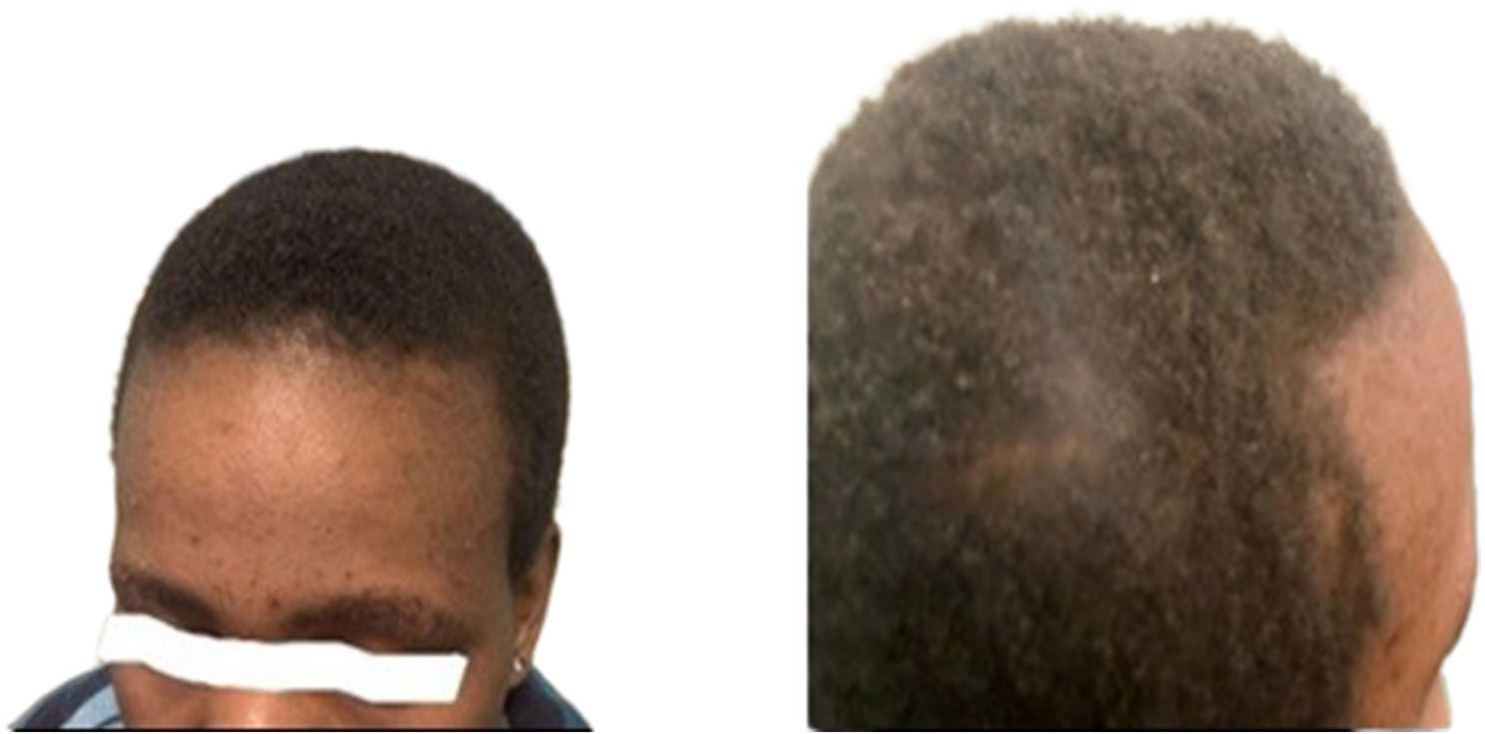
The hair, eyelashes and eyebrows grew back three months after therapy with benzathine Penicillin (pictured here six months after therapy)

**Fig. 4:**
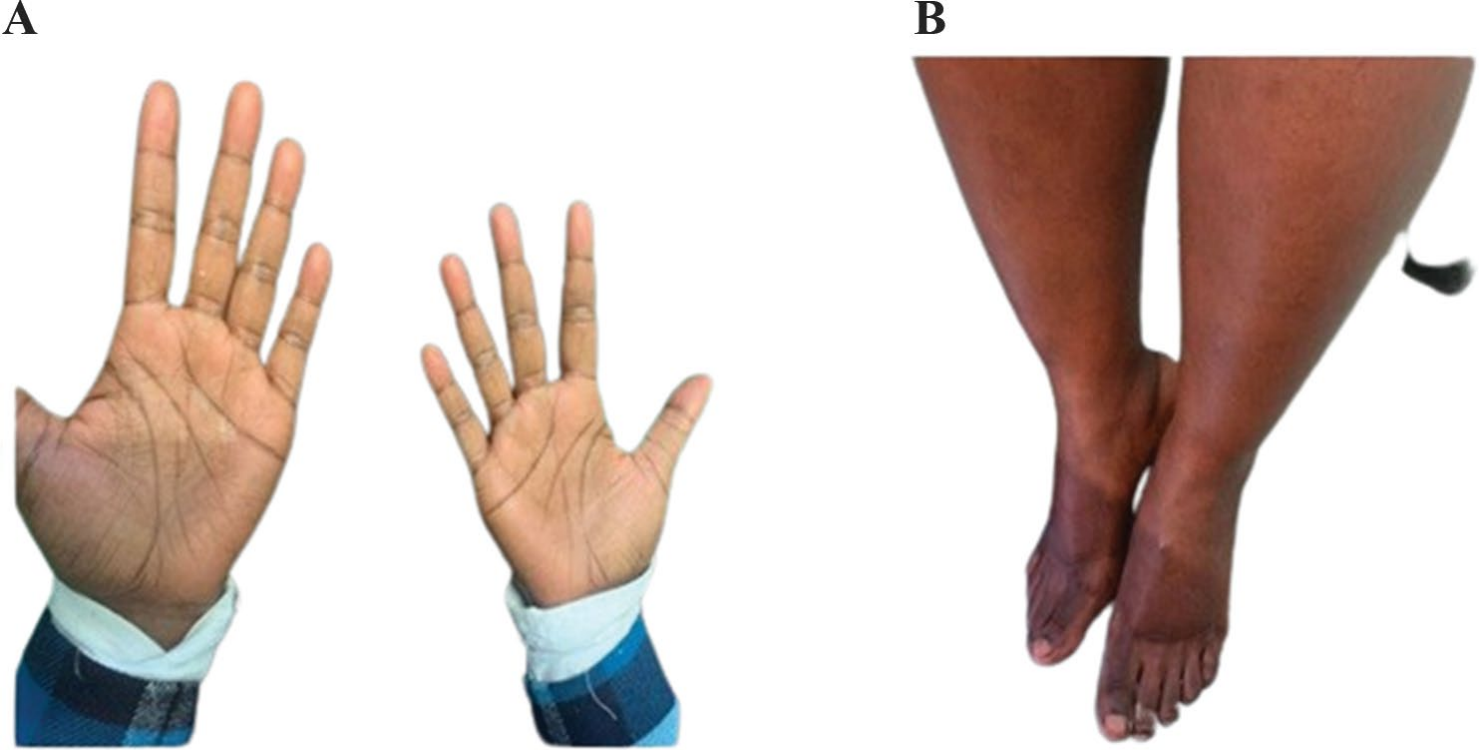
(**A**) The maculopapular lesions disappeared from the palmar surface of both hands within two weeks. (**B**) The hypopigmentation on both feet cleared within three months (pictured here after six months of therapy)

**Fig. 5:**
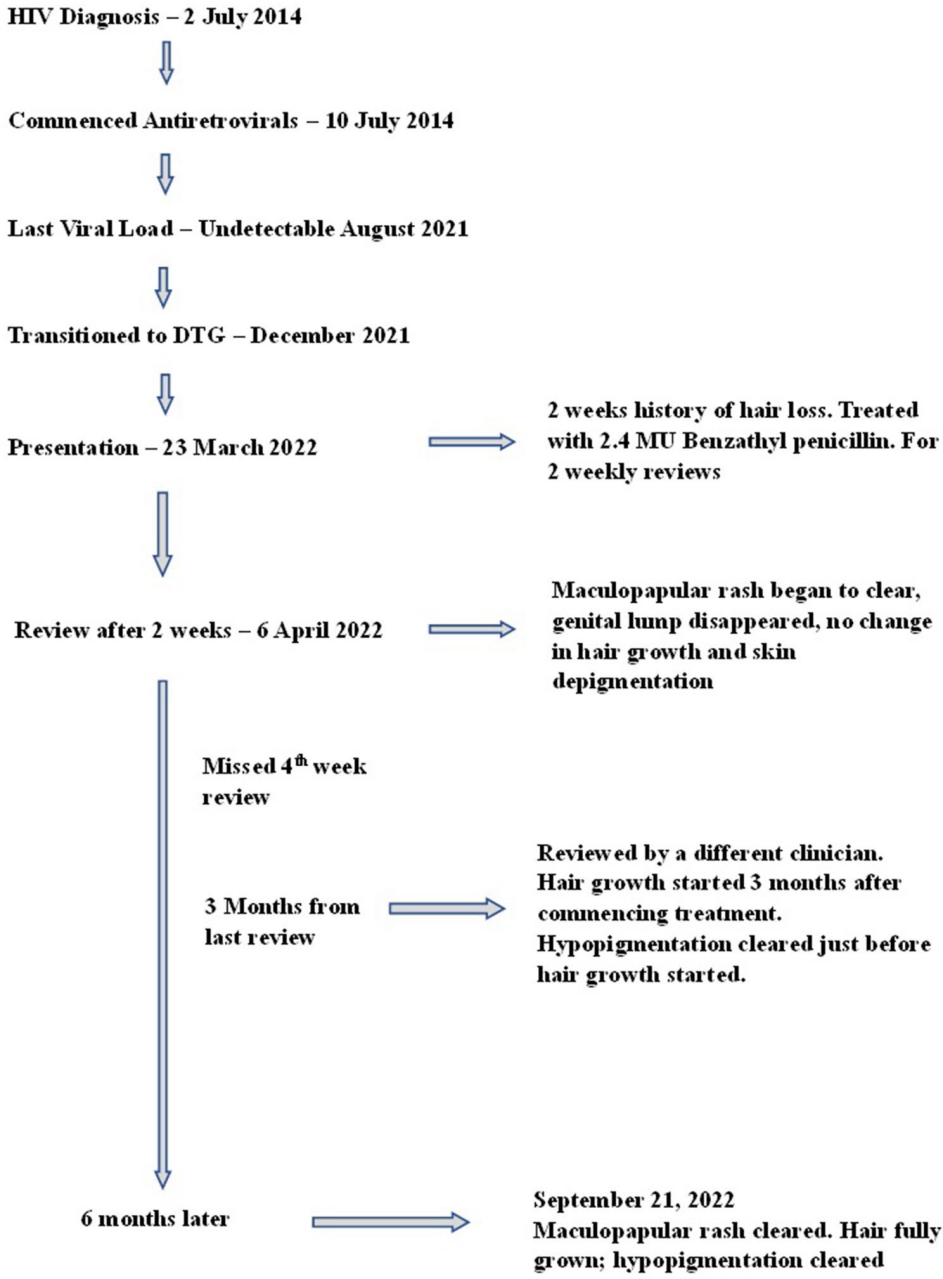
Timelines in patient care from HIV diagnosis to diagnosis of syphilis and treatment

The patient reported that hair started growing three months after treatment, and the hypopigmentation cleared earlier than the hair growth (Figs. 3, 4 and 5).

### Latest patient update

The patient had a viral load taken in August 2022, and it showed that her viral load was elevated at 13,187 copies/ml, and the CD4 count was 675 cells/microlitre. She subsequently admitted that she had struggled to take her treatment since May 2022 due to psychosocial issues. For this, she received stepped-up adherence counselling. By October 2022, she was diagnosed with drug-sensitive TB and had completed treatment. She was further referred to the cardiologist for further review and is being reviewed every six months for symptoms of relapse. In this case, we monitored variables such as RPR titers, clinical symptoms disappearance/relapse, time from treatment to recovery (disappearance of symptoms), and time to relapse, if ever relapse had to reoccur.

### Ethics

The patient gave written informed consent for the pictures to be taken, used for this study, and for reporting clinical details. The Eswatini Health and Human Research Review Board (EHHRB) has approved this case report’s public presentation and dissemination.

## Discussion and conclusions

Our case scenario combines classical features of secondary syphilis with two uncommon features – alopecia and vitiligo, in a client receiving ART. Alopecia syphillitica, also described as “moth-eaten appearance”, is an unusual manifestation of secondary syphilis in 2.9–7% of individuals. It is a non-inflammatory, non-cicatrial hair loss that can present in a diffuse pattern with a classic patchy moth-eaten appearance or a combination of both [13]. The moth-eaten pattern is the most common presentation, as was seen in this case, which also had generalised, patchy hair loss. Two types of syphilis-associated alopecia have been described: symptomatic syphilis alopecia with other cutaneous lesions and the essential type, where no other cutaneous manifestations are noted [14]. The essential type would be complex to diagnose due to the absence of pointers, as seen in our patient with the palmar rash. Histologically, alopecia syphillitica can be differentiated from alopecia areata by the presence of peribulbar eosinophils [15].

Leukoderma syphilliticum, also known as syphilis vitiligo, manifests as hypopigmented patches, usually located in the neck, face and extremities [16]. Very few cases have been reported in the literature, with one previous case reported in an HIV-positive individual [16]. The published cases are heterogeneous in appearance [16]. The original description by Hardy in 1868 pointed to a mottled appearance. Leucoderma syphiliticum encompasses a spectrum of dyschromic lesions that emerge during syphilis [16]. The classic appearance is of small, clear or achromic spots surrounded by pigmented meshes in the early secondary phase [17]. Histologically, there is evidence of melanin absence from the epidermis [18]. In our case, the lesion did affect the extremities (feet), as previously described. However, the quasi-homogeneous presentation of the leukoderma syphilliticum, symmetrically affecting the anterior half of both feet, is particularly unique and could have been vitiligo, pityriasis alba, tinea versicolor, or post-inflammatory hyperpigmentation. However, distinct systemic signs of syphilis, non-atopic dermatological history, and lesion resolution post-treatment seen in our patient increased the odds for syphilis [19]. Acquired epidermodysplasia verruciformis, presenting with erythematous to skin-colored macules, should be considered in HIV-positive patients, as noted in our case, although its persistence and potential for malignant transformation makes it unlikely. Histological examination remains the most reliable diagnostic method for these conditions [19, 20].

The pathogenesis of both leukoderma and syphilitic alopecia is based on an immune response to *Treponema Pallidum*, with resulting loss of terminal hairs, stoppage of the hair cycle and hair bending in the case of alopecia, and melanin loss in the leukoderma [21].

Our client was diagnosed using a syphilis determine test after clinical suspicion. The titres were not measured as the equipment was unavailable. Due to their cost, we did not have access to histology, skin biopsy or other tests that can rule out any cause of alopecia and leukoderma, such as thyroid hormone and auto-immune tests. Therefore, a high level of clinical suspicion is required to avoid incorrect or delayed diagnosis.

The therapeutic approach was based on the WHO guidelines for clients with secondary syphilis, consisting of a stat dose of 2.4 million IU of benzathine penicillin [12]. In the absence of the titres, whether a single dose of benzathine penicillin was sufficient or if the client should receive three doses given as one dose weekly is debatable. The client’s hair started growing 12 weeks post-treatment. Hernández-Bel et al. found in their report of five cases that alopecia disappeared completely within 8 to 12 weeks from treatment [22]. Their population comprised HIV-negative (*n* = 3) and HIV-positive (*n* = 2) clients.

Moreover, their HIV-positive patients received three doses of benzathine penicillin 2.4 million IU, while the HIV-negative clients received a single dose of penicillin. We are uncertain if there is a difference in the time to recovery between our HIV-positive case, who received a single dose of Benzathine Penicillin, and those of Hernández-Bel et al., who received three doses [22]. A larger study would be required to make a definitive conclusion. HIV infection can cause larger or more chancres, accelerated ulcerating secondary Syphilis, frequent ocular Syphilis, and faster progression to late syphilis like neurosyphilis and gummatous syphilis. The former has been reported mostly in those treated for early syphilis with single-dose benzathine penicillin [8]. Within a few weeks or months, leucoderma syphiliticum macules may repigment following therapy with penicillin [16]. In our case, leukoderma had disappeared within three months following Benzathine Penicillin administration. Based on literature and available evidence, the medical team decided the patient should receive two additional doses six months after initiation. Given the numerous reports of neurosyphilis progression after a single dosage of benzathine penicillin, Goh et al. thought in their study that it might be advisable to treat with three doses of benzathine penicillin 2.4 mega units at weekly intervals to avoid such development [8].

This case combined two rare presentations of secondary syphilis in a stable HIV-positive patient. The syphilitic alopecia followed a clinical course that has been commonly reported, but the leukoderma syphilliticum presented with homogeneous lesions in the affected area, contrary to the heterogenous presentation that has been previously described. This observation prompts the need for a high index of suspicion and syphilis investigation for patients with unusual clinical presentations such as vitiligo and alopecia.

## Data Availability

Data used in this case report is available upon a reasonable request to the corresponding author.

## Abbreviations

3TC: Lamivudine
ART: Anti–retroviral therapy
DTG: Dolutegravir
EFV: Efavirenz
EHHRRB: Eswatini Health and Human Research Review Board
FBC: Full Blood Count
HIV: Human Immunodeficiency Virus
RPR: Rapid Plasma Reagin
STI: Sexually Transmitted Infections
TDF: Tenofovir
WHO: World Health Organisation
